# Residues of chlortetracycline, doxycycline and sulfadiazine-trimethoprim in intestinal content and feces of pigs due to cross-contamination of feed

**DOI:** 10.1186/s12917-016-0803-8

**Published:** 2016-09-20

**Authors:** Laura E. J. Peeters, Els Daeseleire, Mathias Devreese, Geertrui Rasschaert, Annemieke Smet, Jeroen Dewulf, Marc Heyndrickx, Hein Imberechts, Freddy Haesebrouck, Patrick Butaye, Siska Croubels

**Affiliations:** 1 Operational Directorate Bacterial Diseases, CODA-CERVA (Veterinary and Agrochemical Research Centre), 1180 Brussels, Belgium; 2 Department of Pathology, Bacteriology and Avian Diseases, Faculty of Veterinary Medicine, Ghent University, 9820 Merelbeke, Belgium; 3 Technology and Food Science Unit, Institute for Agricultural and Fisheries Research, 9090 Melle, Belgium; 4 Department of Pharmacology, Toxicology and Biochemistry, Faculty of Veterinary Medicine, Ghent University, 9820 Merelbeke, Belgium; 5 Veterinary Epidemiology Unit, Department of Reproduction, Obstetrics and Herd health, Faculty of Veterinary Medicine, Ghent University, 9820 Merelbeke, Belgium; 6 Department of Biosciences, School of Veterinary Medicine, Ross University, Basseterre, Saint Kitts and Nevis

**Keywords:** Cross-contamination, Oral bioavailability, Chlortetracycline, Doxycycline, Sulfadiazine, Trimethoprim, Pigs, Intestinal content, Feces

## Abstract

**Background:**

Cross-contamination of feed with low concentrations of antimicrobials can occur at production, transport and/or farm level. Concerns are rising about possible effects of this contaminated feed on resistance selection in the intestinal microbiota. Therefore, an experiment with pigs was set up, in which intestinal and fecal concentrations of chlortetracycline (CTC), doxycycline (DOX) and sulfadiazine-trimethoprim (SDZ-TRIM) were determined after administration of feed containing a 3 % carry-over level of these antimicrobials.

**Results:**

The poor oral bioavailability of tetracyclines resulted in rather high concentrations in cecal and colonic content and feces at steady-state conditions. A mean concentration of 10 mg/kg CTC and 4 mg/kg DOX in the feces was reached, which is higher than concentrations that were shown to cause resistance selection. On the other hand, lower mean levels of SDZ (0.7 mg/kg) and TRIM (< limit of detection of 0.016 mg/kg) were found in the feces, corresponding with the high oral bioavailability of SDZ and TRIM in pigs.

**Conclusions:**

The relation between the oral bioavailability and intestinal concentrations of the tested antimicrobials, may be of help in assessing the risks of cross-contaminated feed. However, future research is needed to confirm our results and to evaluate the effects of these detected concentrations on resistance selection in the intestinal microbiota of pigs.

**Electronic supplementary material:**

The online version of this article (doi:10.1186/s12917-016-0803-8) contains supplementary material, which is available to authorized users.

## Background

Group administration of veterinary drugs through feed and drinking water is frequently applied in the pig industry. Antimicrobials are often administered to pigs by mixing the feed with an oral powder or premix formulation [1–3]. The important role of group administration of antimicrobials in the selection of resistant bacteria is generally recognized [4]. Concerns about antimicrobial resistance selection have already led to the prohibition of use of antimicrobials as growth promoters in Europe since 2006 [5]. However, group medication is still used extensively in many countries for prophylactic, metaphylactic and therapeutic purposes [3]. Major disadvantages of group medication are the poor control over dosage due to differences in feed uptake between sick and healthy animals, inflexible therapy duration for medicated feed, the risk of carry-over and the inevitable contamination of the environment with antimicrobials [4]. Different types of antimicrobial formulations can be used to treat animals in group. Premixes (38.2 %), oral powders (33.7 %) and solutions (19.6 %) each accounted for a significant share of the total amount of sold antimicrobials in 26 European countries in 2013 [1]. However, the types of antimicrobial formulations used for group treatment vary considerably between the individual countries. In some countries, such as Germany, Luxembourg, Estonia and Denmark, it seems that oral powders and solutions are preferred over premix formulations, whereas the opposite applies for countries like Spain, Portugal, Hungary, Cyprus and the UK [1]. In Belgium, both oral powders (70 %, for feed and drinking water) and premixes (20 %) are used frequently [1, 2]. In this study we focus on medicated feed produced in feed mills, and thus on premix formulations.

Carry-over of feed additives and veterinary drugs from a compound feed to a non-target feed is a problem inherent to the production of compound feed in feed mills and the transport, storage and delivery of these feeds [6, 7]. A batch of non-target feed that is produced directly after a compound feed, is generally called ‘flushing feed’. So far, only coccidiostats and histomonostats are included in the European legislation regarding maximum allowed levels (3 %) in flushing feeds [8]. In Belgium, a covenant was established in 2013 between the Belgian Federal Agency for the Safety of the Food Chain (FASFC) and the Belgian Compound Feed Industry Association (BCFIA) [9], in which guidelines for maximum levels of carry-over were set for antimicrobials (1 % of the min. approved dose, except 2.5 % for some formulations in rabbit pellet feed), paracetamol (1 %) and anthelmintics (1–3 % of the max. approved dose, depending on the type of feed). Moreover, due to additional technical requirements for feed mills established in this covenant, namely adding the drugs or additives at the end of the production line instead of the middle, carry-over should be reduced significantly in Belgian feed mills. Unfortunately, carry-over between different feed batches occurs not only in feed mills, but also during transport and at farm level, which makes it a difficult issue to control [10]. A study by Putier et al. [11], investigating carry-over at transport level, indicated that this route should not be underestimated. In this study, two types of carry-over (inter-bin and intra-bin) of antimicrobials (oxytetracycline and chlortetracycline) were measured in ten different types of delivery trucks. Inter-bin carry-over of the two antimicrobials ranged from 0.04 to 1.41 % and intra-bin carry-over ranged from 0 to 0.44 %. Carry-over at farm level remains to be elucidated but could be of great importance, especially in countries with a focus on use of oral powders and solutions as these products are mixed with feed or water at the farm [1].

As a result of cross-contamination of feed, the intestinal microbiota of pigs can be exposed to unintended, low concentrations of antimicrobials [12]. It is known that low antimicrobial concentrations can evoke selection of resistant bacteria in vitro [13, 14] and in vivo [15]. Moreover, in vitro studies with tetracycline, trimethoprim, streptomycin, erythromycin and ciprofloxacin show that the fitness cost for resistance-conferring mutations or genes selected at sub-MIC (Minimum Inhibitory Concentration) concentrations is often lower than for those selected above the MIC [13, 14]. Therefore, these sub-MIC selected mutants would be more stable in bacterial populations and thus potentially more problematic than mutants selected above the MIC [16–18].

In order to assess the true effect of cross-contaminated feed on resistance selection in the intestinal microbiota, it is necessary to first determine the intestinal concentrations of antimicrobials after administration of such feed. Indeed, each type of antimicrobial has different pharmacokinetic (PK) properties that determine the fraction of the orally ingested antimicrobial that remains in the intestines or is excreted in the bile [19]. The oral bioavailability is a measure of the rate and extent of a drug reaching the systemic circulation in its unchanged form through intestinal absorption [20]. As such, this PK property has a significant impact on the fraction of drug that remains in the intestinal content. The oral bioavailability is strongly dependent on the active substance and may be influenced among others by the formulation type and prandial state of the animal. In this study, an in vivo experiment with pigs was set up to determine concentrations in the intestinal content and the feces of chlortetracycline (CTC), doxycycline (DOX) and sulfadiazine-trimethoprim (SDZ-TRIM) when administering feed that contains 3 % of the maximum recommended dose (MRD). This percentage was chosen considering the only legally applicable guideline in Belgium regarding maximum carry-over levels at the time of the experiment (2013) [8]. The choice of antimicrobials was based on two aspects. First, tetracyclines and sulfonamides are among the most used classes of antimicrobials in Belgium when considering oral administration [21]. Second, the oral bioavailability in pigs was taken into account. SDZ, typically used in a combined formulation with TRIM because of the synergistic mode of action, has a very high oral bioavailability in pigs, namely 85–100 % [22, 23]. The same applies to TRIM (73–92 %) [22, 23]. In contrast, tetracyclines have a low oral bioavailability in pigs, with CTC even lower (6 %) than DOX (21–50 %) [24–26].

In the past, studies have been performed to examine levels of antimicrobials and other drugs in tissues and eggs when poultry is fed with cross-contaminated feed [27–29]. Yet no data have been published regarding intestinal concentrations due to cross-contamination in pigs or other livestock. The aim of this study was therefore to determine intestinal concentrations in pigs of CTC, DOX and SDZ-TRIM, when they were fed a diet that contains a 3 % carry-over level of these antimicrobials.

## Methods

### Premixes, reagents and standards

The premixes used for the preparation of the experimental diets were Doxyprex® (active pharmaceutical ingredient, API: 100 mg DOX hyclate/g premix), provided by Kela Veterinaria (Sint-Niklaas, Belgium), Aurofac® (API: 250 mg CTC.HCl/g premix) and Tucoprim® (API: 125 mg SDZ/g premix and 25 mg TRIM/g premix), both provided by Zoetis (Brussels, Belgium). Analytical standards of DOX (doxycycline hyclate), CTC (chlortetracycline.HCl), SDZ and TRIM were obtained from Sigma-Aldrich (Bornem, Belgium). The internal standards (IS) were demethylchlortetracycline.HCl (DMCTC, Sigma-Aldrich) and ^13^C_6_-sulfadimethoxine and d9-trimethoprim, both from Witega (Berlin, Germany). Methanol (CH_3_OH) and acetonitrile (CH_3_CN) were of LC-MS grade and obtained from Biosolve (Valkenswaard, The Netherlands). Water was of LC-MS grade and was obtained from Biosolve (Valkenswaard, The Netherlands) for tetracycline analysis, and was generated from a Milli Q gradient purification system (Millipore, Billerica, MA, U.S.) for SDZ and TRIM analysis. Acetic acid (CH_3_COOH, >99.99 %) was from Sigma Aldrich, succinic acid (C_4_H_6_O_4_) from VWR (Leuven, Belgium) and sodium sulphate (Na_2_SO_4_), formic acid (HCOOH), trichloroacetic acid (CCl_3_COOH) and sodium hydroxide (NaOH) were from Merck (Darmstadt, Germany).

### Preparation of standard solutions

Standard stock solutions of CTC, DOX and the IS DMCTC were prepared in CH_3_OH at a concentration of 1 mg/ml and stored at ≤ −15 °C. Working solutions of DMCTC at a concentration of 100 μg/ml and 20 μg/ml, and of CTC and DOX at a concentration of 100 μg/ml were prepared by appropriate dilution with water. Standard stock solutions of SDZ and the IS ^13^C_6_-sulfadimethoxine were prepared in CH_3_CN/water (50/50, V/V) at a concentration of 1 mg/ml and stored at ≤ −15 °C. Standard stock solutions of TRIM and the IS d9-trimethoprim were prepared in CH_3_OH and stored at ≤ −15 °C. For ^13^C_6_-sulfadimethoxine and d9-trimethoprim, working solutions of 1 μg/ml were prepared in water making use of an intermediate working solution of 10 μg/ml in CH_3_CN/water (50/50, V/V). SDZ and TRIM working solutions of 10 μg/ml, 1 μg/ml and 0.1 μg/ml were prepared in water and used for spiking the calibration samples. To prepare sodium succinate 0.1 M, 11.8 g of C_4_H_6_O_4_ was dissolved in 600 ml of water, 10 M NaOH was added until pH 4.0 was reached, and water was added to obtain a final volume of 1000.0 ml. The solution was stored at 4.0 °C. Solutions of HCOOH (0.1 %), CCl_3_COOH (20 %) and CH_3_COOH (0.1 %) were prepared by appropriate dilutions with water.

### Preparation of the experimental feed

Three different batches of experimental diets were prepared. Blank feed (meal n° 9231, AVEVE, Merksem, Belgium) was mixed with the DOX, CTC and SDZ-TRIM premixes, respectively. A custom made mixing device (Silobouw, Zulte, Belgium) was kindly provided by the Food Science and Technology Unit of Ghent University. The added amounts of antimicrobials were calculated to yield cross-contamination levels in the feed corresponding to 3 % of the MRD (CTC, 18.6 mg/kg BW (body weight)/day; DOX, 13.5 mg/kg BW/day; SDZ, 25.0 mg/kg BW/day; TRIM, 5.0 mg/kg BW/day). BW and daily feed intake were set at 25 and 1.5 kg respectively. Thus, a target concentration of 9.29 mg CTC/kg feed, 6.76 mg DOX/kg feed, 12.5 mg SDZ/kg feed and 2.50 mg TRIM/kg feed was aimed for. Each premix was first mixed manually with 10 kg of blank feed, which was then mixed with the remaining blank feed (120 kg) in the feed mixer for 25 min. The feed was collected from the mixer in 13 bags, each containing 10 kg. One sample of approximately 200 g was taken from bag n° 1, 2, 3, 5, 6, 8, 9, 11, 12 and 13 of each experimental feed to assess if the target concentration was achieved and to determine the homogeneity. In this way, samples were collected at the beginning, middle and at the end of the mixing stream, in order to monitor the whole mixing cycle. The samples were kept at room temperature (t_R,_ 15–25 °C) until analysis (within a time frame of 2 weeks).

### Animal experiment

Twenty-four pigs with an average BW of 27.0 ± 4.0 kg were randomly divided into 4 equal groups (3 males and 3 females/group): one control group and three experimental groups. Each group was housed in a strictly separated 3 by 4 m pen with a concrete floor and natural light cycle. The temperature varied between 21 and 25 °C. The floor was cleaned with water every day just before sample collection. Ad libitum access to drinking water and feed was provided throughout the experiment. After a 1-week acclimatization period, each experimental group received during ten days experimental feed containing 3 % cross-contamination levels of either CTC, DOX or SDZ-TRIM. The control group received blank feed (no antimicrobials). Individual fecal samples were taken by rectal stimulation, just before the start of providing the experimental diets and at day 2, 4, 6, 8 and 10 of the experimental feeding period. In case no individual sample could be obtained (which was the case for in total 19 time points from 12 pigs), fresh fecal samples were collected from the cleaned floor. On day 11, all animals were euthanized through a combined IM injection of xylazine (4.4 mg/kg BW), zolazepam and tiletamine (both 2.2 mg/kg BW) followed by an intracardial injection of 0.3 ml/kg BW of T61® (MSD, Brussels, Belgium). Immediately after euthanasia, samples of cecal content and colonic content from different sampling segments [proximal colon ascendens (PCA), distal colon ascendens (DCA), colon descendens (CD)] were taken from each animal individually. The samples were directly stored at −80 °C without homogenization.

### Quantitation of antimicrobials in feed and feces

In-house developed methods were applied for both analysis of tetracyclines [30, 31] and SDZ-TRIM [27, 28].

- Tetracyclines analysis
  Twenty-five ml of CH_3_OH were added to 5.0 g of each feed sample. After 20 min of shaking on an in-house rotary shaker, samples were centrifuged (6261 *g*, 10 min, 4 °C). Two-hundred μl of supernatant were transferred into an Eppendorf tube and 800 μl of CH_3_OH were added. After adding 50 μl of IS (20 μg/ml), samples were vortex mixed. Next, the samples were filtered (PVDF 0.22 μm Millex-GV, Millipore, Overijse, Belgium) and transferred to an autosampler vial and 5 μl was injected onto the LC-MS/MS instrument.
  To 2.0 g of intestinal content or feces, 50 μl of IS (100 μg/ml) were added. After vortex mixing (15 s), 10.0 ml of sodium succinate solution (0.1 M) were added and the samples were again vortex mixed (15 s). Samples were then shaken (20 min, in-house rotary shaker) and centrifuged (6261 *g*, 10 min, 4 °C). The supernatant was transferred to a new plastic tube and vortex mixed (15 s) after adding 1.0 ml of 20 % CCl_3_COOH. These tubes were centrifuged again (6261 *g*, 10 min, 4 °C) and the samples were filtered through a Whatman filter (Whatman n°541, VWR, Leuven). This filtrate was used for further solid-phase clean-up. After preconditioning an OASIS® HLB 60 mg/3 ml solid phase extraction column (Waters, Milford, MA, U.S.) with consecutively 3 ml of CH_3_OH, 3 ml of HCl (1 M) and 3 ml of HPLC water, the filtrate was poured onto the HLB column. The column was then washed with 1 ml of water and dried. The analytes were eluted with 3 ml of CH_3_OH. The eluate was passed through a PVDF filter, transferred to an autosampler vial and 5 μl was injected onto the LC-MS/MS instrument.
  The LC system consisted of an Acquity autosampler and an Acquity binary solvent manager from Waters (Milford, U.S.). Chromatographic separation was achieved on an Acquity UPLC BEH C18 column (50 mm × 2.1 mm i.d., 1.7 μm) from Waters. The temperatures of the autosampler tray and column oven were set at 10 °C and 30 °C, respectively. Mobile phase A consisted of CH_3_CN whereas mobile phase B was 0.1 % HCOOH in water. Flow rate was set at 0.3 ml/min and the following elution program was run: 0–4.0 min (10 % A), 4.0–5.0 min (linear gradient to 90 % A), 5.0–7.1 (90 % A), 7.1–7.2 (linear gradient to 10 % A), 7.2–9.0 min (10 % A). The detection was performed with a Quattro Premier XE triple quadrupole mass spectrometer, equipped with an electrospray ionization (ESI) probe operating in the positive ionization mode (Waters). Masslynx software v 4.1 was used to quantitate, based on the following MS-MS transitions: *m/z* 479.04 > 461.84 (CTC) and *m/z* 445.10 > 427.96 (DOX).
- Sulfadiazine-trimethoprim analysis
  After homogenization of the feed sample, 5.0 g of feed was weighed and 50 μl of each IS (1 mg/ml) and 25 ml of CH_3_OH were added. The sample was vortex mixed, shaken on a horizontal shaker (Edmund Bühler, Hechingen, Germany) during 30 min, and centrifuged (4000 *g*, 15 min, t_R_). Five ml of the supernatant were evaporated to dryness at 45 ± 5 °C under nitrogen. The sample was redissolved in 10 ml of CH_3_CN/water (50/50 V/V), vortex mixed (30 s), diluted to 1/15 in CH_3_CN/water (50/50, V/V), vortex mixed (30 s) and transferred to an autosampler vial.
  For intestinal content or feces analysis, 2.0 g of sample was weighed after homogenization and 40 μl of each IS (1 μg/ml) were added. The sample was carefully mixed with 8 g of Na_2_SO_4_ with a spatula to obtain a dry mixture. If necessary, extra Na_2_SO_4_ was added until the sample was dry. After adding 10 ml of CH_3_CN, the sample was vortex mixed, shaken during 30 min (horizontal shaker, Edmund Bühler) and centrifuged (15 min, 4000 *g,* t_R_). Five ml of the supernatant were then transferred into a glass tube and evaporated to dryness under nitrogen in a water bath of 45 °C. Next, the sample was redissolved in 1 ml of an CH_3_CN/water mixture (50/50, V/V) containing 0.1 % CH_3_COOH in water, vortex mixed (30 s), sonicated (5 min), and filtered through a PVDF filter into an autosampler vial.
  Chromatographic separation was performed on a Waters Acquity UPLC system. An Acquity UPLC BEH C18 column (100 mm × 2.1 mm i.d., 1.7 μm) was used and the analysis was performed with a gradient of water/CH_3_CN (95/5, V/V) + 0.3 % CH_3_COOH (mobile phase A) and water/CH_3_CN (5/95, V/V) + 0.3 % CH_3_COOH (mobile phase B). The following elution program was run: 0–8 min (100 % A), 8–12 min (70 % A), 12–13 min (0 % A), 13–13.01 min (linear gradient to 100 % A), 13.01–14.6 min (100 % A). Flow rate was set at 0.4 ml/min. A Xevo TQ-MS triple quadrupole mass spectrometer with an ESI probe operating in the positive ionization mode was used. Quantitation was done with Masslynx software v 4.1. MS-MS transitions for SDZ were: *m/z* 250.89 > 155.94/107.96 and for TRIM: *m/z* 290.98 > 122.99/230.01. The detected ion ratio’s for the different samples were within the permitted tolerances specified in Commission Decision 2002/657/EC [32].

### Method validation

The methods were validated for feed and feces according to a set of parameters that were in compliance with the recommendations and guidelines defined by the European Community [32] and international standards for validation of analytical methods in residue depletion studies [33]. The following set of parameters was determined: limit of detection (LOD, *n* = 6), limit of quantification (LOQ, *n* = 6), linearity (R^2^ and goodness-of-fit coefficient (g)), precision (repeatability, RSD_r_ (*n* = 6), and reproducibility, RSD_R_ (*n* = 6)) and trueness (*n* = 6). Validation samples were prepared with blank feed from the same batch as the feed that was administered during the experiment and blank feces were obtained from pigs that were not treated with antimicrobial drugs.

### Statistical analysis

After determination of normality and homogeneity of variances, one-way analysis of variance (ANOVA) (SPSS 22, IBM, Chicago, IL, U.S.) was performed for each antimicrobial on the concentrations from the four different intestinal segments. A Scheffé test was performed as post-hoc test. The significance level was set at 0.05.

## Results

### Method validation

The results of the method validation are given in Additional file 1: Table S1. All values, except for the trueness in case of SDZ in feed, were within the acceptance ranges according to Commission Decision 2002/657/EC [32].

### Concentrations in experimental feed

Ten samples of each batch of experimental feed were analyzed to assess if the target concentrations (3 % of the MRD) were achieved. Mean concentrations ± standard deviation (SD) in the feed were 7.33 ± 6.87 mg/kg (=3.26 % of MRD) for DOX, 9.98 ± 5.35 mg/kg (=3.23 % of MRD) for CTC, 12.99 ± 4.15 mg/kg (=3.12 % of MRD) for SDZ and 2.31 ± 0.90 mg/kg (=2.77 % of MRD) for TRIM. In all experimental diets, there was a high variation between sample concentrations.

### Concentrations in feces

**Fig. 1:**
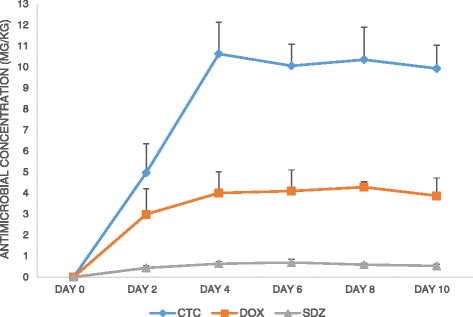
Mean concentrations (mean of six pigs + standard deviation) of chlortetracycline (CTC), doxycycline (DOX) and sulfadiazine (SDZ) in pig feces during 10 days of ad libitum feeding with feed containing 3 % cross-contamination levels of these antimicrobials. For CTC, concentrations rose from 4.97 mg/kg to a steady-state of approximately 10 mg/kg. Mean concentrations of DOX rose from 2.99 mg/kg to a steady-state of approximately 4 mg/kg. Mean concentrations of SDZ rose from 0.44 mg/kg to a steady-state of approximately 0.70 mg/kg

The mean (+ SD) concentrations of CTC, DOX and SDZ in the feces are shown in Fig. 1. A steady-state was reached around day 4 for CTC (±10 mg/kg), DOX (±4 mg/kg) and SDZ (±0.7 mg/kg). Concentrations of TRIM were very low; all results except two were lower than the LOD of 0.016 mg/kg. No traces of antimicrobials were found in the fecal samples taken on day 0, just before the start of the experimental period.

Transfer ratio’s (TR), i.e. the mean concentration in feces taken over day 4 – day 10 of the experimental period, and divided by the mean concentration in feed, were 102.5, 55.4 and 4.7 % for CTC, DOX and SDZ, respectively (Table 1).

**Table 1:** Oral bioavailability (F) compared to transfer ratio’s (TR) of chlortetracycline (CTC), doxycycline (DOX) and sulfadiazine (SDZ)

Antimicrobial	F	TR				
		CM	PCA	DCA	CD	Feces
CTC	6 % [25]	69.6 %	84.2 %	71.4 %	101.0 %	102.5 %
DOX	21–50 % [24, 26]	24.3 %	45.1 %	36.6 %	51.9 %	55.4 %
SDZ	85–100 % [22, 23]	3.7 %	3.5 %	5.2 %	4.2 %	4.7 %

TR’s are calculated by dividing the mean concentration (6 pigs) in the content of an intestinal segment (*CM* caecum, *PCA* proximal colon ascendens, *DCA* distal colon ascendens, *CD* colon descendens) after 10 days of feeding by the mean concentration in the feed. The mean concentration in the feces was taken over day 4 - day 10 (steady-state)

### Concentrations in cecal and colonic content

**Fig. 2:**
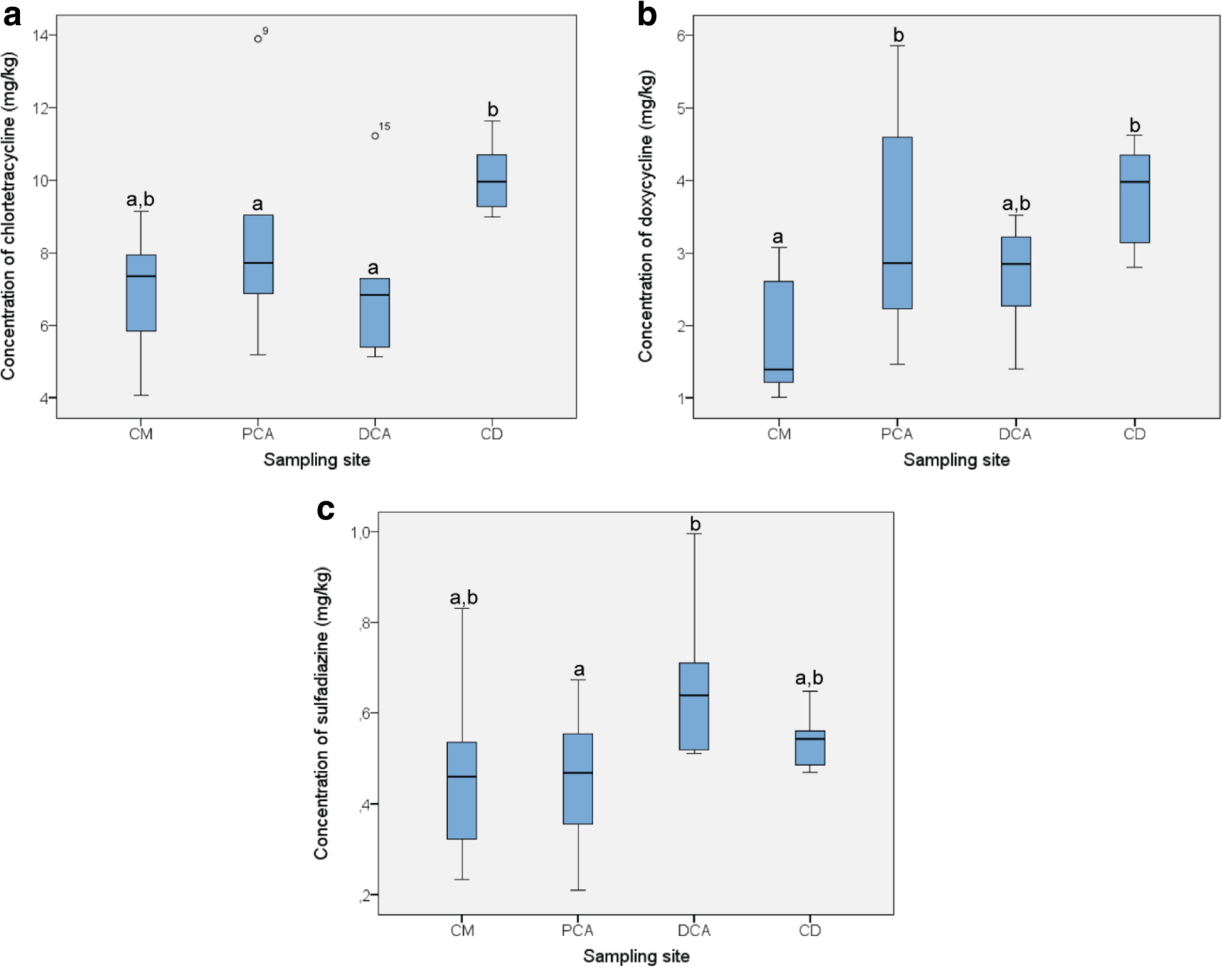
Concentrations of A) chlortetracycline (CTC), B) doxycycline (DOX) and C) sulfadiazine (SDZ) in cecal content and colonic content from three different sampling segments (6 indepentent observations per sampling segment). Samples were taken from 6 pigs per group after 10 days of ad libitum feeding with feed containing 3 % cross-contamination levels of CTC, DOX and SDZ. Mean concentrations in caecum (CM), proximal colon ascendens (PCA), distal colon ascendens (DCA) and colon descendens (CD) were 6.95, 8.41, 7.12 and 10.08 mg/kg (CTC), 1.78, 3.31, 2.68 and 3.81 mg/kg (DOX) and 0.47, 0.45, 0.67 and 0.54 mg/kg (SDZ), respectively. The two outlying values for CTC (observation 9 and 15) belong to one animal. A different letter (a or b) denotes a significant difference in concentration between sampling segments (*p* < 0.05)

CTC, DOX and SDZ concentrations in cecal content and contents of different segments of the colon after 10 days of feeding are presented in Fig. 2. Min/max/mean concentrations for CTC in the different intestinal segments were 4.06/9.15/6.95 mg/kg (caecum, CM), 5.20/13.89/8.41 mg/kg (PCA), 5.14/11.22/7.12 mg/kg (DCA) and 8.99/11.63/10.08 mg/kg (CD). For DOX, these concentrations were 1.01/3.07/1.78 mg/kg (CM), 1.47/5.86/3.31 mg/kg (PCA), 1.40/3.51/2.68 mg/kg (DCA) and 2.80/4.62/3.81 mg/kg (CD). SDZ concentrations were 0.23/0.83/0.47 mg/kg (CM), 0.21/0.67/0.45 mg/kg (PCA), 0.51/1.00/0.67 mg/kg (DCA) and 0.47/0.65/0.54 mg/kg (CD). All results for TRIM were again lower than the LOD of 0.016 mg/kg. Concentrations in the CD approached the average feces concentration found for CTC, DOX and SDZ. CTC concentrations found in the CD proved to be significantly higher compared to concentrations in the DCA and the CM, but not compared to the PCA. DOX levels in the CM were significantly lower than in the PCA and CD. SDZ levels in the DCA were significantly higher than in the PCA. The TR’s, i.e. the mean concentration in CM, PCA, DCA or CD divided by the mean concentration in the feed, are given in Table 1.

## Discussion

The aim of this study was to determine which concentrations of CTC, DOX, SDZ and TRIM could be found in intestinal content and feces of pigs when feed containing a 3 % carry-over concentration was administered.

In each experimental diet the target concentration of 3 % of the MRD was approached (2.77–3.26 %). Although the best possible sampling procedure [7] was applied, a large variation between the samples was found. Adequate mixing of the premixes depends on multiple factors, including physico-chemical properties such as particle size and electrostatic properties of the premix. Other factors that influence homogeneity are the composition of the final feed, type of mixing machinery, mixing in stages or trituration and precision and size of the samples taken for analysis [34]. Since very small volumes of premix had to be mixed with large amounts of blank feed, it is not surprising that a large variability among samples was found. Moreover, studies on cross-contamination in feed mills show that antimicrobials are not homogenously divided in flushing feed either [6]. In contrast, concentrations found in intestinal content and feces showed a much lower variation.

In case of SDZ-TRIM, concentrations found in feces and intestinal content were very low (SDZ max. 0.995 mg/kg, TRIM < LOD). Except for two values, all results for TRIM were lower than the LOD. Since the administered dose was very low (2.31 mg/kg feed), the reported oral bioavailability for TRIM is high (73–92 % [22, 23]) and elimination occurs through renal excretion, very low intestinal concentrations were indeed expected. On the other hand, quantitative results for SDZ were obtained, although the oral bioavailability of SDZ in pigs is even higher than for TRIM. This can be explained by the higher absolute dosage of SDZ (12.99 mg/kg feed) compared to TRIM, as both compounds are present in a 5/1 ratio (SDZ/TRIM) in the used formulation. Interestingly, the calculated TR’s from feed to gut of SDZ (3.5–5.2 %) correspond well to the high oral bioavailability reported in pigs (85–100 % [22, 23]), i.e. the higher the oral bioavailability the lower residual concentrations in the gut can be expected unless extensive biliary excretion or secretion in the gut takes place.

The concentrations of tetracyclines in feces and cecal and colonic content were found to be relatively high. In general, higher concentrations were seen in the distal part of the colon compared to the proximal part and the caecum. The main explanation for these observations is probably the progressive absorption of water throughout the intestinal tract. As in the case of SDZ, the calculated TR’s from feed to gut (CTC 69.6–102.5 %, DOX 24.3–55.4 %) correspond well to the reported oral bioavailability in pigs (CTC 6 %, DOX 21–50 % [24–26]). It has to be taken into account though, that the bioavailability of tetracyclines is highly variable, most likely due to presence of feed in the gastrointestinal tract. It is known that oral absorption of tetracyclines may be reduced in the presence of bivalent ions [24, 35]. Also, the study design to calculate oral bioavailabilities may vary between different studies, e.g. the prandial state of the animals. Taking into account that our experiment involves feed administration, references regarding oral bioavailability in non-fasted pigs in particular were consulted. Especially for DOX, the oral bioavailability seems to vary, even within the same study between individual animals (8.0–32.4 % [24], 40–50 % [26]). The intestinal concentrations of DOX in the present study correspond best to the oral bioavailability reported in a previous study that also used a premix formulation (40–50 %) [26] when compared to administration of an oral powder (8–32.4 %) [23]. Besides oral bioavailability, also the excretion route can influence the intestinal concentrations of a drug. SDZ-TRIM and CTC are renally excreted whereas up to 75 % of DOX is excreted unchanged in the intestinal tract [36]. It would therefore be expected that the TR ratio of DOX is higher than based solely on oral bioavailability. The large variability in reported oral bioavailabilities for DOX might explain the relatively low TR indicating that this TR only serves as a guidance value and depends on several factors.

The oral bioavailability of a drug is usually determined for its therapeutic dose. Given the inverse relation found between the oral bioavailability and intestinal concentrations of SDZ, CTC and DOX, it is rather likely that there is a linear relation between the administered dose and intestinal concentrations. This information can be used in the risk assessment of different cross-contamination levels of pig feed regarding potential resistance selection in the intestinal microbiota. However, this conclusion can only be drawn for the tested antimicrobials and animal species. Furthermore, additional experiments should be performed to confirm our results - ideally testing a range of antimicrobial concentrations - as there is no previous research available to compare. A non-peer reviewed report [37] though, estimated intestinal concentrations of CTC to be 1.68 mg/kg in case of administration of 12 mg CTC/kg feed. This is clearly lower compared to our results (min 5.1–max 13.9 mg/kg CTC in colonic content with 9.98 mg CTC/kg feed).

In a recent study [12], manure samples obtained from different pig, poultry and veal calve farms in the Netherlands were examined for the presence of antimicrobial residues. In 16 out of 20 of the sampled pig farms, residues were detected although no recent use of antimicrobials was reported. Tetracyclines were found in 14 of these farms, with DOX concentrations ranging from 2 to 95,000 μg/kg. Sulfonamides were detected in 6 out of 20 farms, with SDZ concentrations ranging from 1 to 216 μg/kg. In light of these data, it is clear that one should not focus on the absolute results based on 3 % carry-over levels obtained in this study, but rather on the relation found between the oral bioavailability of CTC, DOX and SDZ and intestinal concentrations.

In recent years, more attention has been paid to the possible effects of low antimicrobial concentrations on selection of resistant bacteria. Pioneer studies revealed important effects of very low concentrations on resistance selection in vitro. Gullberg et al. performed competition experiments between strains resistant and susceptible to tetracycline and found minimal selective concentrations of 15 ng/ml (competition between isogenic - except for the resistance determinant - *Salmonella* Typhimurium strains) [13] and 45 ng/ml tetracycline (competition between isogenic *E. coli* strains, with or without resistance plasmid pUUH239.2) [14]. The minimal selective concentration was in this case defined as the concentration where the fitness cost of the resistance is balanced by the antimicrobial-conferred selection for the resistant mutant. This would mean that even concentrations of tetracyclines 100× lower than those found in this study can cause resistance selection. Brewer et al. [15] investigated the effect of 1 μg/ml of different antimicrobials on transfer of resistance genes in vivo in pigs and found that 1 μg/ml of tetracycline and sulfamethazine increased transfer frequency, whereas 1 μg/ml of sulfathiazole did not. It is likely that intestinal concentrations of 1 μg/ml of tetracyclines can be found in pigs, considering our results and the maximum allowed carry-over level (1 %) established in the Belgian covenant [9].

## Conclusions

This study showed an inverse relation between intestinal concentrations and the oral bioavailability for SDZ-TRIM as well as for CTC and DOX, which have a high, respectively low oral bioavailability in pigs. As different studies [6, 12] indicate there is a large variation in cross-contamination levels of feed, this result can be an important tool to evaluate possible risks of different contamination levels. Further research is needed to determine the effect on resistance selection in the intestinal microbiota. Furthermore, it would be interesting to perform additional experiments, confirming our results and analyzing other antimicrobials that are frequently used as premix formulation, such as penicillins, macrolides and polymyxins [2].

## Additional file

Additional file 1: Table S1. Validation parameters for quantification of chlortetracycline (CTC), doxycycline (DOX), sulfadiazine (SDZ) and trimethoprim (TRIM) in pig feed and feces. (DOCX 15 kb)

## Abbreviations

API: Active pharmaceutical ingredient
BW: Body weight
C_4_H_6_O_4_: Succinic acid
CCl_3_COOH: Trichloroacetic acid
CD: Colon descendens
CH_3_CN: Acetonitrile
CH_3_COOH: Acetic acid
CH_3_OH: Methanol
CM: Caecum
DCA: Distal colon ascendens
DMCTC: Demethylchlortetracycline.HCl
DOX: Doxycycline
HCOOH: Formic acid
IS: Internal standard
LOD: Limit of detection
LOQ: Limit of quantitation
MIC: Minimum inhibitory concentration
MRD: Maximum recommended dose
Na_2_SO_4_: Sodium sulphate
PCA: Proximal colon ascendens
PK: Pharmacokinetic
SD: Standard deviation
SDZ: Sulfadiazine
t_R_: Room temperature
TR: Transfer ratio
TRIM: Trimethoprim
