# Supplementary material for: Residues of chlortetracycline, doxycycline and sulfadiazine-trimethoprim in intestinal content and feces of pigs due to cross-contamination of feed
Source: BMC Vet Res. 2016 Sep 20;12:209. doi: 10.1186/s12917-016-0803-8 (PMC5028959; doi:10.1186/s12917-016-0803-8)
Supplement: Additional file 1: Table S1. — Validation parameters for quantification of chlortetracycline (CTC), doxycycline (DOX), sulfadiazine (SDZ) and trimethoprim (TRIM) in pig feed and feces. (DOCX 15 kb) [file 12917_2016_803_MOESM1_ESM.docx]

| **Table S1. Validation parameters for quantification of chlortetracycline (CTC), doxycycline (DOX), sulfadiazine (SDZ) and trimethoprim (TRIM) in pig feed and feces** | | | | | | | | | |
| --- | --- | --- | --- | --- | --- | --- | --- | --- | --- |
| **Matrix** | **Analyte** | **Validation levels (feed: mg/kg; feces: µg/kg)** | **LOD (feed: mg/kg; feces: µg/kg) N=6** | **LOQ (feed: mg/kg; feces: µg/kg) N=6** | **Linearity (R^2^)** | **Goodness-of-fit coefficient (g)** | **RSD_r_ (%)**  **N=6** | **RSD_R_ (%)**  **N=6** | **Trueness (%)**  **N=6** |
| Feed | CTC | 4.65 / 9.29 / 11.63 | 0.47 | 4.65 | 0.9959 | 3.96 | 12.2 / 6.9 / n.d. | n.d. / n.d. / 4.0 | 96.1 / 85.9 / 99.1 |
|  |  |  |  |  |  |  |  |  |  |
|  | DOX | 3.61 / 6.76 | 0.09 | 3.61 | 0.9974 | 2.95 | 9.3 / 8.7 | n.d. | 95.4 / 89.4 |
|  |  |  |  |  |  |  |  |  |  |
|  | SDZ | 7.0 / 15.0 | 1.0 | 2.0 | 0.9946 | 9.00 | 8.1 / 4.0 | n.d. | 139.9* / 117.9* |
|  |  |  |  |  |  |  |  |  |  |
|  | TRIM | 7.0 / 15.0 | 0.25 | 0.5 | 0.9968 | 19.0 | 2.6 / 1.9 | n.d. | 97.0 / 105.4 |
|  |  |  |  |  |  |  |  |  |  |
| Feces | CTC | 1,000 / 4,500 | 34.4 | 1,000 | 0.9989 | 2.96 | 6.6 / 3.9 | n.d. / 6.3 | 98.2 / 105.4 |
|  |  |  |  |  |  |  |  |  |  |
|  | DOX | 1,000 / 4,500 | 11.5 | 1,000 | 0.9959 | 6.11 | 6.5 / 2.9 | n.d. / 10.6 | 106.4 / 103.2 |
|  |  |  |  |  |  |  |  |  |  |
|  | SDZ | 100 / 250 / 500 | 33.0 | 67.0 | 0.9929 | 19.0 | 9.1 / 11.5 / 5.1 | 8.6 / 11.4 / 7.6 | 94.6 / 97.9 / 98.0 |
|  |  |  |  |  |  |  |  |  |  |
|  | TRIM | 100 / 250 / 500 | 16.0 | 32.0 | 0.9947 | 13.0 | 7.2 / 6.3 / 2.7 | 6.1 / 8.1 / 6.4 | 100.7 / 101.1 / 102.5 |
|  |  |  |  |  |  |  |  |  |  |
| Acceptance criteria [1]: trueness 80-110%; RSD_r max_: 4.65 mg/kg: 14.1%, 9.29 mg/kg: 12.7% (CTC, after dilution); 3.61 mg/kg: 12.7%, 6.76 mg/kg: 11.5% (DOX, after dilution),7.0 mg/kg: 7.9 %, 15.0 mg/kg: 7.1% (SDZ, TRIM), 1,000 µg/kg: 10.7%, 4,500 µg/kg: 8.5% (CTC, DOX), 100 µg/kg: 15.1%, 250 µg/kg: 13.1%, 500 µg/kg: 11.9% (SDZ, TRIM); RSD_R max_: 11.63 mg/kg: 11.1% (CTC); 4,500 µg/kg: 12.8% (CTC, DOX), 100 µg/kg: 22.6%, 250 µg/kg: 19.7%, 500 µg/kg: 17.8% (SDZ, TRIM)  LOD, limit of detection; LOQ, limit of quantification; RSD_r_, repeatability, RSD_R_, within laboratory reproducibility; n.d., not determined  *: criterium not fulfilled | | | | | | | | | |

1. Commission Decision 2002/675/EC implementing Council Directive 96/23/EC concerning the performances of analytical methods and the interpretation of results. Off. J. European Union 2002, L221:8–36
